# Prognosis Prediction of Colorectal Cancer Using Gene Expression Profiles

**DOI:** 10.3389/fonc.2019.00252

**Published:** 2019-04-09

**Authors:** Feixia Pan, Tianhui Chen, Xiaohui Sun, Kuanrong Li, Xiyi Jiang, Asta Försti, Yimin Zhu, Maode Lai

**Affiliations:** ^1^Department of Epidemiology & Biostatistics, School of Public Health, Zhejiang University, Hangzhou, China; ^2^Sir Run Run Shaw Hospital, College of Medicine, Zhejiang University, Hangzhou, China; ^3^Group of Molecular Epidemiology & Cancer Precision Prevention, Institute of Occupational Diseases, Zhejiang Academy of Medical Sciences, Hangzhou, China; ^4^First Affiliated Hospital of Wenzhou Medical University, Wenzhou, China; ^5^Guangzhou Women and Children's Medical Center, Guangzhou Medical University, Guangzhou, China; ^6^Division of Molecular Genetic Epidemiology, German Cancer Research Center (DKFZ), Heidelberg, Germany; ^7^Key Laboratory of Disease Proteomics of Zhejiang Province, Department of Pathology, School of Medicine, Zhejiang University, Hangzhou, China

**Keywords:** colorectal cancer, prognostic index, gene expression, prognosis prediction, combined predictor

## Abstract

**Background:** Investigation on prognostic markers for colorectal cancer (CRC) deserves efforts, but data from China are scarce. This study aimed to build a prognostic algorithm using differentially expressed gene (DEG) profiles and to compare it with the TNM staging system in their predictive accuracy for CRC prognosis in Chinese patients.

**Methods:** DEGs in six paired tumor and corresponding normal tissues were determined using RNA-Sequencing. Subsequently, matched tumor and normal tissues from 127 Chinese patients were assayed for further validation. Univariate and multivariate Cox regressions were used to identify informative DEGs. A predictive index (PI) was derived as a linear combination of the products of the DEGs and their Cox regression coefficients. The combined predictive accuracy of the DEGs-based PI and tumors' TNM stages was also examined by a logistic regression model including the two predictors. The predictive performance was evaluated with the area under the receiver operating characteristics (AUCs).

**Results:** Out of 75 candidate DEGs, we identified 10 DEGs showing statistically significant associations with CRC survival. A PI based on these 10 DEGs (PI-10) predicted CRC survival probability more accurately than the TNM staging system [AUCs for 3-year survival probability 0.73 (95% confidence interval: 0.64, 0.81) vs. 0.68 (0.59, 0.76)] but comparable to a simplified PI (PI-5) using five DEGs (LOC646627, BEST4, KLF9, ATP6V1A, and DNMT3B). The predictive accuracy was improved further by combining PI-5 and the TNM staging system [AUC for 3-year survival probability: 0.72 (0.63, 0.80)].

**Conclusion:** Prognosis prediction based on informative DEGs might yield a higher predictive accuracy in CRC prognosis than the TNM staging system does.

## Introduction

Colorectal Cancer (CRC) is one of the most common malignancies globally (1). In order to guide clinical treatment and predict prognosis, several CRC staging systems have been established, especially the American Joint committee on Cancer (AJCC) tumor-node-metastasis (TNM) system based on anatomical information, which is widely used (2). According to the TNM staging system, the survival of CRC patients is related to the size of primary tumor (T), nearby lymph nodes affected (N), and distant metastasis (M). However, CRC is an etiologically heterogeneous disease involving several distinct biologic pathways, resulting in different survival status even among patients who are at the same TNM stage (3).

Over last few decades we have seen a remarkable advance in the knowledge of CRC biological pathways with an abundance of novel molecular biomarkers having been found to have potentials in prognosis prediction. By applying the quantitative reverse transcription polymerase chain reaction (RT-qPCR) platform, O'Connell et al. selected seven recurrence risk genes among patients with stage II/III colon cancer and developed a recurrence risk score using the seven genes to stratify patients with significantly different recurrence risks (4). Barrier et al. also reported an 80% prognosis prediction accuracy obtained by profiling 30 genes among stage II colon cancer patients (5). Regarding the overall survival, it has been reported that molecular staging based on 43 core genes was 90% accurate in predicting 36-month overall survival, significantly better than Dukes' staging (6). Investigation on prognostic markers for CRC deserves efforts, but data from China are scarce.

The objective of the present study was to build a prognostic index (PI) based on differentially expressed gene (DEG) profiles between tumor and normal tissues and to compare this PI with the TNM staging system regarding their accuracy in prognosis prediction among Chinese CRC patients.

## Materials and Methods

### Flowchart of This Study and DEG Selection

**Figure 1:**
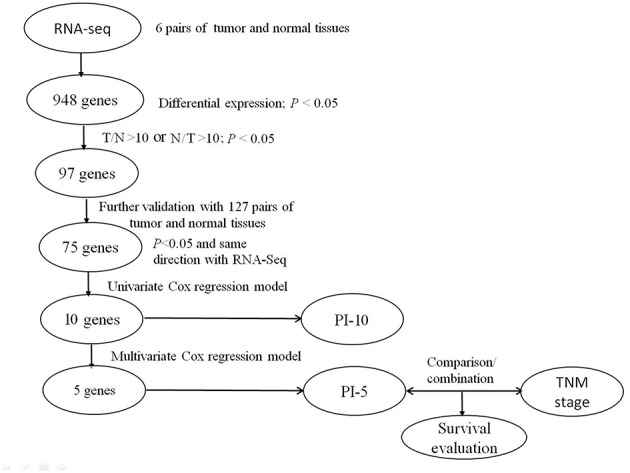
Flowchart of the study.

As shown in the flowchart (Figure 1), tumor-normal matched tissue samples of CRC were collected at the time of surgery and immediately stored in liquid nitrogen. We applied the RNA-Sequencing (RNA-Seq) approach to identify candidate DEGs among six pairs of tumor and corresponding normal tissues (5 cm from the edge of the tumor). RNA was extracted following the instruction of RNeasy Plant Mini Kit (QIAGEN Inc., Valencia, CA, USA). The total RNA of all the samples was first treated with DNase I to degrade any possible DNA contamination. The mRNA was then enriched using oligo (dT) magnetic beads and mixed with a fragmentation buffer to be fragmented into approximately 200-bp fragments. First-strand cDNA synthesis was performed using random hexamers. Buffer, dNTPs, RNase H, and DNA polymerase I were added to synthesize the second strand. The double-stranded cDNA was purified with magnetic beads. End preparation and 3′-end addition of the nucleotide adenine (A) were performed. Finally, sequencing adaptors were ligated to the fragments. The fragments were enriched by PCR amplification. During the QC step, the Agilent 2100 Bioanalyzer and ABI StepOnePlus Real-Time PCR System were used to qualify and quantify the DNA library. The library products were then sequenced with the Illumina HiSeq 2000.

The levels of gene expressions were calculated using the reads per kilobase million (RPKM) method. Using the method proposed by Audic and Claverie (7), we identified 97 candidate DEGs (differentiated expression ≥10 folds, *P* < 0.05) from 948 genes (Supplementary Table S1).

### Patients and Tumor Samples

Afterwards, we verified the 97 DEGs with the QuantiGene Plex assay performed on 127 pairs of tumor and matched normal tissues. We recruited 127 patients (82 men) diagnosed with CRC and received resection between September 2006 and February 2012. All tumor samples were collected before any systemic chemotherapy. The main patient and tumor characteristics are shown in Table 1. Clinically relevant data, including socio-demographic and pathological information (sex, age, tumor location, tumor size, depth of tumor invasion, lymph node metastasis, distant metastasis, TNM stage, and postoperative chemotherapy), were collected by reviewing the medical records. We eventually identified 75 DEGs (*P* < 0.05 and same direction as in RNA-Seq) for further analyses. This study was approved by the ethics committee of Zhejiang University and all the patients provided a written informed consent.

**Table 1:** The main patient and tumor characteristics, stratified by 3-year survival status.

**Variables**	**N**	**Survivors**	**Non-survivors**	*P*-value^*^
**GENDER**				
Male	82	60	22	0.071
Female	42	24	18	
**AGE (YEARS)**				
≤60	46	35	11	0.127
>60	78	49	29	
**LOCATION**				
Rectum	71	47	24	0.670
Colon	53	37	16	
**MAXMUM DIAMETER**				
≤5	80	50	30	0.098
>5	40	31	9	
**TNM STAGE**				
I	18	16	2	0.091
II	26	19	7	
III	50	29	21	
IV	26	16	10	
**DEPTH OF TUMOR INVASION (T)**				
T1-T3	60	47	13	0.007
T4	60	33	27	
**LYMPH NODE METASTASIS (N)**				
N0	55	43	12	0.016
N1-N2	66	38	28	
**DISTANT METASTASIS (M)**				
M0	97	67	30	0.466
M1	26	16	10	
**POSTOPERATIVE CHEMOTHERAPY**				
No	48	32	16	0.839
Yes	76	52	24	

* *Univariate analysis of categorical variables was performed using χ^2^*.

### Statistical Analysis

We used univariate and multivariate Cox proportional hazards models to explore the associations between the identified DEGs and the overall survival time after resection. The multivariate model adjusted for sex, age, TNM stage, postoperative chemotherapy, and DEGs. A PI was derived as a linear combination of the products of the DEGs and their coefficients obtained from the univariate and multivariate Cox regressions. All DEGs were mean-centered to ensure that PI of zero corresponds to the survival probability given that all the DEGs are at their medium level, with PI < 0 and PI > 0 indicating the good and poor prognosis, respectively. The predictive performance of the PI was investigated with the area under the receiver operating characteristics (ROC) curves. DEGs-based PI grade was then established according to the cut-off value which maximizes the Youden's index. Furthermore, we developed combined predictors (CPs) for prognosis prediction (1-year, 3-year, and 5-year survival) using logistic regression, which included both the DEGs-based PI grade and tumors' TNM stages. The accuracy of CPs, DEGs-based PI grade and TNM staging system for prognosis prediction was compared by the area under the ROC curve (AUC). Additionally, we used the indicator of category-free net reclassification improvement (cfNRI) to evaluate the effect of prognosis prediction for DEGs-based PI grade.

Statistical analyses were performed using the SAS statistical software, version 9.3 (SAS Institute, Cary, NC, USA) and R version 3.2.2 (R Foundation for Statistical Computing, Vienna, Austria). Two-sided *P*-value < 0.05 was considered statistically significant.

## Results

**Figure 2:**
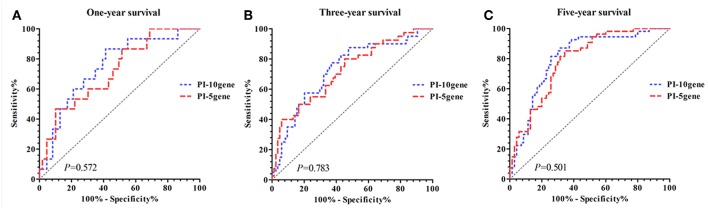
Comparison of predictive performance for PI-10 and PI-5. **(A)** PI-10 vs. PI-5 (1-year survival). **(B)** PI-10 vs. PI-5 (3-year survival). **(C)** PI-10 vs. PI-5 (5-year survival).

After controlling for sex, age, TNM stage and postoperative chemotherapy in the multivariate Cox regression model, 10 out of the 75 DEGs showed statistically significant associations with the overall survival time. As shown in Table 2, CPNE8, LOC646627, CDKN2A, ATP6V1A, SCARA5, BEST4, and KLF9 were positively associated with the overall survival time, while DNMT3B, GRIN2D, and ANLN were negatively associated with the overall survival. By summing up the products of the 10 DEGs and their Cox regression coefficients, we developed a PI (hereinafter referred to as PI-10), which ranged from −6.280 to 5.694, with the quartiles being −0.956, 0.118, and 1.057, respectively. The ROC curves for PI-10 to predict 1-year, 3-year, and 5-year survival are given in Figures 2A–C (blue line), and the AUCs were 0.748, 0.730, and 0.807, respectively.

**Table 2:** DEGs statistically significantly associated with the overall survival time.

**Gene name**	**β^a^**	**SE**	***P*-value**
UNIVARIATE COX MODEL^*^			
CPNE8	0.365	0.109	0.001
LOC646627	0.285	0.124	0.022
DNMT3B	−0.756	0.278	0.007
CDKN2A	0.329	0.134	0.014
ATP6V1A	0.450	0.155	0.004
SCARA5	0.260	0.117	0.026
ANLN	−0.343	0.170	0.044
BEST4	0.289	0.108	0.008
KLF9	0.237	0.114	0.037
GRIN2D	−0.443	0.207	0.033
MULTIVARIATE COX MODEL^*^			
LOC646627	0.263	0.123	0.032
BEST4	0.246	0.102	0.016
KLF9	0.412	0.122	0.001
ATP6V1A	0.613	0.162	0.001
DNMT3B	−0.832	0.291	0.004

* *Univariate and multivariate Cox proportional hazard regression models were adjusted by sex, age, TNM, and postoperative chemotherapy*.

a *PI was derived as a linear combination of the products of the DEGs and their coefficients obtained from the Cox models, with PI < 0 and PI > 0 indicating the good and poor prognosis, respectively*.

To shrink the number of DEGs involved in prognosis prediction, we performed a multivariate Cox regression on the 10 DEGs, sex, age, TNM, stage and postoperative chemotherapy, which ended up with 5 independent DEGs, i.e., LOC646627, BEST4, KLF9, ATP6V1A, and DNMT3B (Table 2). Thus, we developed a parsimonious PI based on these 5 DEGs (hereinafter referred to as PI-5). In comparison with PI-10, PI-5 yielded improved AUCs for all the three survival intervals of interest (0.720, 0.722, and 0.790, respectively), which however was not statistically significant (Figures 2A–C, red line). No significant difference of AUC was found between PI-10 and PI-5.

**Figure 3:**
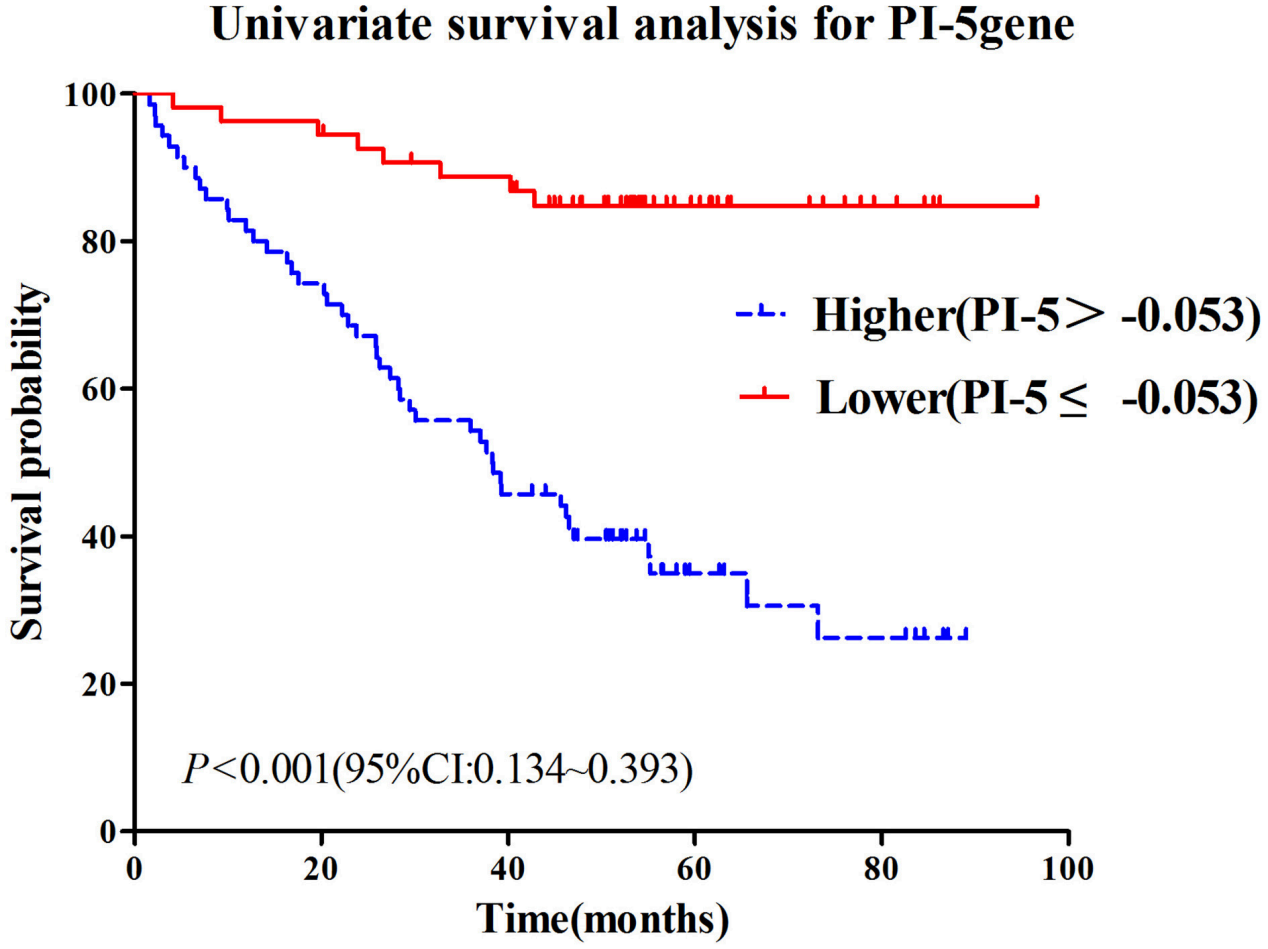
Survival time and survival probability by tumor grade as defined using PI-5: high grade *vs*. low grade.

For PI-5, a cut-off point of −0.053 would maximize the Youden's index, reaching 0.344, 0.348, and 0.509 for all the three survival intervals of interest, respectively (Supplementary Table S2 and Supplementary Figure S1). Subsequently, we categorized the patients into two groups: high grade (PI-5 > −0.053) and low grade (PI-5 ≤ −0.053). The survival probabilities for the patients with low grade were statistically significantly higher than those with high grade (Figure 3). The survival time for patients with low grade and high grade was 85.77 ± 3.59 vs. 45.52 ± 3.92 (Supplementary Table S3). We further derived combined predictive indexes (CPIs) from logistic models in which PI-5 grade and TNM stage were both included as predictor variables, as shown below: CPI for 1-year survival = PI-5 + 0.301^*^TNM; CPI for 3-year survival = PI-5 + 0.235^*^TNM; CPI for 5-year survival = PI-5 + 0.199^*^TNM.

**Figure 4:**
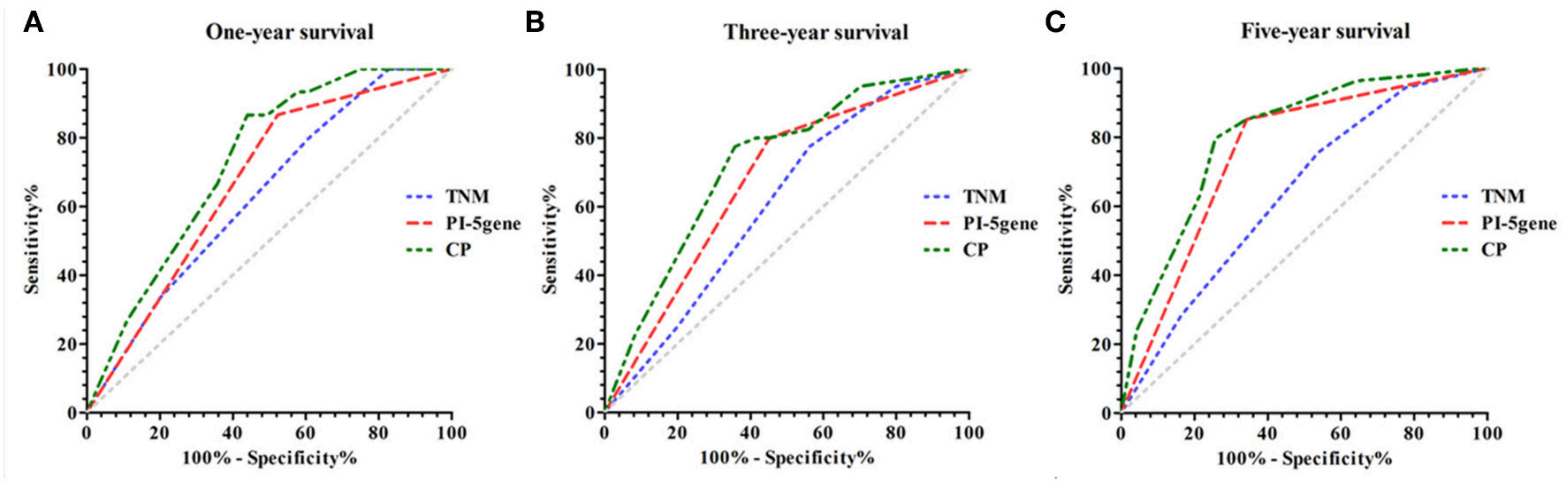
Comparison of predictive performance for PI-5, TNM stage and CPI. **(A)** 1-year survival. **(B)** 3-year survival. **(C)** 5-year survival.

Figure 4 and Supplementary Table S4 compare the AUCs among PI-5 grade, TNM stage, and CPI, consistently showing significantly higher AUC for CPI than for PI-5 grade and TNM stage across the 3-year, and 5-year survival intervals (*P* < 0.05). Specifically, PI-5 grade yielded an improvement in the AUC compared to TNM stage for all the three survival intervals of interest, yet no significant difference was observed for the 1-year and 3-year survival. The AUCs for 1-year, 3-year, and 5-year survival of PI-5 grade and TNM stage were 0.676 vs. 0.634, 0.681 vs. 0.611, and 0.760 vs. 0.637, respectively. Moreover, CPI showed significantly higher AUCs compared to TNM stage, for 3-year and 5-year intervals of interest (*P* < 0.05), reaching 0.719 and 0.801, respectively (referring to AUCs elevation of 17.68 and 25.75%, respectively). Additionally, the cfNRIs (0.295, 0.391, and 0.464 for the three survival intervals, respectively) showed significantly improved predictions by PI-5 grade for all (Supplementary Table S5).

## Discussion

Among a pool of 75 DEGs, we identified 10 DEGs that showed statistically significant associations with CRC survival after surgery. Additionally, we developed a PI based on 5 DEGs, which performed better than the classical TNM staging system for CRC prognosis prediction. We found that it is worthwhile to combine the DEGs-based PI and the long-established TNM staging system given significantly improved predictive accuracy gained by doing so.

The DEGs which we identified in the present study to have statistically significant associations with the survival probability of CRC patients after surgery confirms the findings of previous studies which suggested gene expression profiling to improve accuracy of prognosis prediction (8–10). From the genes included in the PI-5, it has been found that BEST4 is a member of the bestrophin gene family (BEST1, BEST2, BEST3, and BEST4) of anion channels. The BEST4 was predominantly expressed in the colon and weakly in fetal brain, spinal cord, retina, lung, trachea, testis and placenta. Significantly down regulation of BEST2 was found in the active lesions of ulcerative colitis. In contrast to BEST2, the expression of BEST4 appeared to be maintained (11). So far, there was little to no research on the mechanism of BEST4 contributing to the development of CRC. In the present study, significant down regulation of BEST4 was found in tumor issues of CRC patients. However, we observed a statistically negative association between BEST4 expression and the survival probability of CRC patients after surgery, suggesting that the role of this gene in CRC prognosis merits further investigation.

DNMT1, DNMT3A, and DNMT3B are the major DNA methyltransferases (DNMTs) that so far have been found in mammals. An established body of knowledge concludes that DNA hypomethylation plays a crucial role in human cancers (12). DNMT3B has been reported to be overexpressed in breast, oral, and colorectal tumor tissues (13–15), while other studies have suggested that DNMT3B and DNMT3A are tumor suppressor genes for lymphoma and lung cancer (16, 17). In the present study, however, we observed a positive association between DNMT3B expression and survival probability among CRC patients after surgery, inconsistent to what we expected, therefore we assume that DNMT3B is likely to have a tumor suppressing effect in colorectal carcinogenesis.

LOC646627 encodes LYPD8 protein, a family member of LY6/PLAUR. LYPD8 can mediate segregation of intestinal bacteria and epithelia cells in the colon to preserve intestinal homeostasis (18). In the present study, LYPD8 was underexpressed in the tumor tissues and was associated with poor prognosis. Chronic inflammation targets the intestinal microbiota and impacts the progression of CRC by inducing the expansion of microbes including *E. coli*, which has carcinogenic effect (19). For CRC patients after resection, intestinal homeostasis can moderate the inflammatory response and thus prevent the occurrence of complications following surgery.

In the present study, we observed an overexpression of ATP6V1A in CRC tumor tissues, which had an adverse effect on prognosis. The ATP6V1A gene encodes a component of vacuolar ATPase (V-ATPase), an enzyme that mediates the acidification of eukaryotic intracellular organelles. Studies have reported overexpressed ATP6V1A in gastric tumor issues and its association with cancer prognosis, suggesting that ATP6V1A might be a target of gastric cancer treatment (20). However, studies investigating the ATP6V1A expression in other tumor tissues are scarce.

It has been reported that KLF9 exhibited low expression in pancreatic cancer, and upregulation of KLF9 may inhibit the progression of pancreatic cancer (21). However, the expression of KLF9 was up-regulated in human ovarian cancer, and KLF9 deficiency significantly inhibited tumor growth in nude mice (22). What was more, some results show KLF9 to be haploinsufficient suppressor of colon tumorigenesis in the ApcMin/+mouse colon by suppressing expression of ISG15, an apoptosis-inhibiting cytokine (23). Contrary to what we found in the present study, KLF9 was low expressed in CRC tumor tissues and was associated with poor prognosis.

The TNM staging system has been widely adopted for prognosis prediction and treatment strategy selection. This staging system relies solely on anatomical information about the size and extent of primary tumor. Since more and more novel promising non-anatomical prognostic factors have been identified, the TNM staging system calls for an evolution so as to remain usable in the era of personalized diagnosis and molecular-targeted therapy. As a response to this need, two genetic biomarkers, namely KRAS gene mutation and 18q loss of heterozygosity, along with other five factors, have been incorporated into the 7th revision of the TNM staging system (24), though the resulting improvement in predictive capacity compared with its predecessor is disputable (25, 26).

The present study is one of the few studies that aimed to build a PI by integrating informative genetic biomarkers. We found that this DEGs-based PI predicted the survival probability among CRC patients after resection more accurately than the classic TNM staging system (AUC for five-year survival probability 0.77 vs. 0.65) and comparable to most of the reported CRC prognosis prediction nomograms based on non-DEGs data (27). A recent study reported that a multi-RNA-based classifier also outperformed the TNM staging system regarding the overall survival (AUC 0.83 vs. 0.74) (28). However, our results still supported the predictive value of the TNM staging system.

The strength of the present study includes its comprehensive search for statistically informative DEGs and thus it provides important insights into their value in clinical decision-making process. However, some limitations of the present study should be noted. First of all, our study was moderate in its sample size and therefore unbiased estimates of model coefficients were difficult to achieve. Secondly, like many other studies, we did not validate our DEGs-based PI externally and its performance may thus be subject to over-optimism.

In conclusion, this study confirms that prognosis prediction based on informative DEGs might yield a higher accuracy than the TNM staging system alone. Therefore, we recommend integration of differentially expressed gene data into the TNM staging system for further improvement in CRC prognosis prediction.

## Ethics Statement

This study was approved by the ethics committee of Zhejiang University and all the patients provided a written informed consent.

## Author Contributions

ML and YZ are responsible for the study concept and design. YZ and TC obtained funding. YZ and FP acquired data, analyzed, and interpreted data. KL, XJ, and TC drafted the manuscript, and all authors revised it for important intellectual content. YZ and TC are the guarantors of this work. All co-authors commented the manuscript and approved the submission.

### Conflict of Interest Statement

The authors declare that the research was conducted in the absence of any commercial or financial relationships that could be construed as a potential conflict of interest.

## Abbreviations

CRC: colorectal cancer
DEG: differentially expressed gene
PI: predictive index
ROC: receiver operating characteristics
AUC: area under the curve
TNM: tumor-node-metastasis
CPI: combined predictive index
cfNRI: category-free net reclassification improvement.
