# Supplementary material for: Prognosis Prediction of Colorectal Cancer Using Gene Expression Profiles
Source: Front Oncol. 2019 Apr 9;9:252. doi: 10.3389/fonc.2019.00252 (PMC6465763; doi:10.3389/fonc.2019.00252)
Supplement: Supplementary file 1 [file Data_Sheet_1.docx]

**Supplementary Table S1** 97 candidate DEGs

| Gene name | Cancer tissue* | Normal tissue* | *P* value |
| --- | --- | --- | --- |
| ABCA8 | 0.07(0.04~0.17) | 0.63(0.32~1.40) | <0.001 |
| ABI3BP | 0.03(0.01~0.10) | 0.31(0.14~0.58) | <0.001 |
| ABLIM2 | 0.03(0.01~0.07) | 0.00(0.00~0.01) | <0.001 |
| ADAMDEC1 | 0.06(0.03~0.12) | 0.30(0.15~0.62) | <0.001 |
| ADH1A | 0.20(0.04~0.64) | 1.82(1.27~2.52) | <0.001 |
| ADHFE1 | 0.01(0.00~0.01) | 0.03(0.02~0.05) | <0.001 |
| AHCY | 1.78(0.96~3.12) | 0.31(0.26~0.43) | <0.001 |
| ANLN | 0.84(0.49~1.45) | 0.18(0.10~0.34) | <0.001 |
| ANPEP | 0.20(0.09~0.58) | 1.08(0.61~1.84) | <0.001 |
| AQP8 | 0.02(0.01~0.15) | 0.82(0.25~2.76) | <0.001 |
| BEST4 | 0.02(0.01~0.06) | 0.88(0.48~2.30) | <0.001 |
| CA1 | 0.04(0.01~0.20) | 2.69(0.98~6.03) | <0.001 |
| CA7 | 0.01(0.01~0.03) | 0.37(0.21~0.57) | <0.001 |
| CNR1 | 0.02(0.01~0.05) | 0.18(0.08~0.57) | <0.001 |
| CPNE8 | 0.03(0.01~0.05) | 0.05(0.00~0.10) | <0.001 |
| ETV4 | 0.47(0.24~0.90) | 0.03(0.01~0.18) | <0.001 |
| FAM189A2 | 0.01(0.01~0.02) | 0.06(0.03~0.19) | <0.001 |
| FGL2 | 0.05(0.03~0.14) | 0.34(0.22~0.54) | <0.001 |
| FOXF2 | 0.02(0.01~0.03) | 0.07(0.04~0.16) | <0.001 |
| GAB3 | 0.03(0.01~0.06) | 0.13(0.06~0.30) | <0.001 |
| GAS5 | 1.20(0.86~1.78) | 0.75(0.47~0.97) | <0.001 |
| GPM6B | 0.01(0.01~0.03) | 0.06(0.02~0.10) | <0.001 |
| GRIN2D | 0.34(0.14~0.57) | 0.09(0.04~0.38) | <0.001 |
| HPRT1 | 0.35(0.20~0.61) | 0.14(0.10~0.30) | <0.001 |
| KIAA1199 | 0.50(0.19~1.21) | 0.06(0.04~0.10) | <0.001 |
| KLF9 | 0.27(0.16~0.50) | 0.68(0.48~0.91) | <0.001 |
| LOC646627 | 0.10(0.03~0.31) | 0.86(0.38~1.87) | <0.001 |
| MAL | 0.05(0.02~0.13) | 0.48(0.22~1.36) | <0.001 |
| MET | 0.67(0.35~1.09) | 0.06(0.03~0.10) | <0.001 |
| MT1M | 0.25(0.10~0.82) | 2.50(1.40~3.68) | <0.001 |
| NOVA1 | 0.03(0.01~0.09) | 0.24(0.12~0.52) | <0.001 |
| P2RX1 | 0.06(0.03~0.13) | 0.43(0.31~0.71) | <0.001 |
| PDK4 | 0.18(0.09~0.39) | 0.93(0.47~1.37) | <0.001 |
| PKNOX2 | 0.01(0.00~0.02) | 0.05(0.03~0.09) | <0.001 |
| PLP1 | 0.01(0.00~0.05) | 0.21(0.11~0.40) | <0.001 |
| PPAP2A | 0.44(0.33~0.68) | 1.19(0.93~1.46) | <0.001 |
| PPM1H | 0.15(0.09~0.25) | 0.04(0.03~0.10) | <0.001 |
| PTN | 0.05(0.03~0.09) | 0.15(0.09~0.23) | <0.001 |
| RNF112 | 0.03(0.01~0.06) | 0.16(0.07~0.56) | <0.001 |
| RSPO2 | 0.06(0.02~0.15) | 0.48(0.24~1.01) | <0.001 |
| S100B | 0.07(0.04~0.14) | 0.33(0.18~0.81) | <0.001 |
| S100P | 4.23(1.93~6.99) | 0.40(0.18~0.69) | <0.001 |
| SCARA5 | 0.01(0.00~0.03) | 0.14(0.08~0.22) | <0.001 |
| SCNN1B | 0.10(0.04~0.29) | 0.78(0.55~1.10) | <0.001 |
| SEMA3D | 0.03(0.01~0.05) | 0.14(0.09~0.39) | <0.001 |
| SEMA6D | 0.06(0.03~0.14) | 0.34(0.22~0.48) | <0.001 |
| SFRP1 | 0.01(0.01~0.03) | 0.14(0.07~0.29) | <0.001 |
| SLC6A6 | 0.39(0.22~0.69) | 0.12(0.08~0.18) | <0.001 |
| SOX9 | 0.70(0.39~0.99) | 0.25(0.14~0.52) | <0.001 |
| SQLE | 1.07(0.44~1.69) | 0.20(0.14~0.28) | <0.001 |
| SRPX | 0.04(0.02~0.09) | 0.25(0.10~0.45) | <0.001 |
| SULT2B1 | 0.26(0.12~0.44) | 0.05(0.02~0.17) | <0.001 |
| TRIB3 | 0.65(0.32~1.36) | 0.23(0.09~0.85) | <0.001 |
| TSPAN11 | 0.02(0.01~0.03) | 0.05(0.03~0.09) | <0.001 |
| ZNF256 | 0.03(0.01~0.06) | 0.06(0.04~0.19) | <0.001 |
| AZGP1 | 0.48(0.19~0.80) | 0.12(0.04~0.38) | <0.001 |
| TET2 | 0.22(0.15~0.28) | 0.30(0.26~0.39) | <0.001 |
| TKT | 4.65(2.45~6.85) | 1.00(0.85~1.33) | <0.001 |
| ABCB1 | 0.10(0.04~0.24) | 0.17(0.08~0.43) | <0.001 |
| CDKN2A | 0.05(0.02~0.15) | 0.01(0.00~0.03) | <0.001 |
| TET1 | 0.02(0.01~0.05) | 0.10(0.03~0.35) | <0.001 |
| RARB | 0.08(0.04~0.17) | 0.23(0.09~0.71) | <0.001 |
| PLOD3 | 0.49(0.27~0.72) | 0.10(0.02~0.16) | <0.001 |
| TET3 | 0.37(0.26~0.58) | 0.21(0.16~0.33) | <0.001 |
| MLH1 | 0.28(0.21~0.39) | 0.17(0.11~0.22) | <0.001 |
| CLU | 0.13(0.07~0.26) | 0.80(0.24~1.50) | <0.001 |
| TCF3 | 0.92(0.57~1.38) | 0.50(0.36~0.75) | <0.001 |
| DNMT3A | 0.30(0.22~0.48) | 0.17(0.05~0.24) | <0.001 |
| DNMT1 | 0.54(0.35~0.80) | 0.27(0.16~0.39) | <0.001 |
| TGM2 | 0.45(0.25~0.76) | 0.19(0.00~0.44) | <0.001 |
| DUSP22 | 0.06(0.04~0.10) | 0.14(0.07~0.37) | <0.001 |
| MGMT | 0.18(0.12~0.29) | 0.09(0.04~0.12) | <0.001 |
| WFDC2 | 0.16(0.04~0.38) | 0.05(0.00~0.12) | <0.001 |
| ATP6V1A | 0.18(0.13~0.24) | 0.09(0.00~0.14) | <0.001 |
| DNMT3B | 0.09(0.04~0.21) | 0.05(0.03~0.12) | <0.001 |
| C2CD4A | 0.05(0.02~0.11) | 0.07(0.02~0.33) | 0.020 |
| CDCA3 | 0.24(0.13~0.36) | 0.16(0.05~0.41) | 0.048 |
| CDH3 | 0.51(0.26~0.82) | 0.22(0.06~3.48) | 0.033 |
| CHI3L1 | 0.14(0.07~0.38) | 0.13(0.03~0.43) | 0.095 |
| CLDN1 | 0.38(0.19~0.83) | 0.17(0.04~0.87) | 0.001 |
| FOXQ1 | 0.10(0.03~0.22) | 0.04(0.01~0.34) | 0.057 |
| JPH1 | 0.35(0.21~0.54) | 0.22(0.12~0.58) | 0.035 |
| KIF14 | 0.13(0.08~0.18) | 0.07(0.03~0.23) | 0.003 |
| KIF20A | 0.26(0.15~0.40) | 0.22(0.06~0.83) | 0.767 |
| KIF4A | 0.03(0.02~0.05) | 0.05(0.01~0.31) | 0.027 |
| KRT80 | 0.45(0.25~0.79) | 0.47(0.16~1.70) | 0.493 |
| MEX3A | 0.14(0.08~0.30) | 0.18(0.07~0.72) | 0.080 |
| MSLN | 0.34(0.06~1.07) | 0.30(0.14~0.70) | 0.957 |
| PAQR4 | 0.25(0.15~0.45) | 0.20(0.06~0.80) | 0.278 |
| PDX1 | 0.17(0.06~0.36) | 0.06(0.02~0.36) | <0.001 |
| PHLDA1 | 0.25(0.13~0.42) | 0.16(0.07~0.43) | 0.009 |
| PVRL4 | 0.13(0.07~0.20) | 0.05(0.03~0.18) | <0.001 |
| RHPN1 | 0.16(0.11~0.35) | 0.16(0.07~0.57) | 0.352 |
| SIM2 | 0.16(0.05~0.30) | 0.05(0.01~0.32) | 0.005 |
| SLCO4A1 | 0.18(0.09~0.32) | 0.11(0.04~0.55) | 0.093 |
| TACSTD2 | 0.08(0.04~0.35) | 0.07(0.02~0.30) | 0.024 |
| TRIM29 | 0.54(0.19~1.15) | 0.28(0.11~0.85) | 0.005 |

*DEGs expression was shown as median(P25~P75 interquartile interval).

**Supplementary Table S2** Sensitivity and specificity of different PI-5gene cut-off points for prognosis prediction

| PI-5gene | One year | | | Three year | | | Five year | | |
| --- | --- | --- | --- | --- | --- | --- | --- | --- | --- |
| Cut-off | Sensitivity | specificity | Youden | Sensitivity | specificity | Youden | Sensitivity | specificity | Youden |
| -0.568 | 1.000 | 0.294 | 0.294 | 0.900 | 0.333 | 0.233 | 0.963 | 0.429 | 0.392 |
| -0.549 | 1.000 | 0.303 | 0.303 | 0.900 | 0.345 | 0.245 | 0.963 | 0.443 | 0.406 |
| -0.531 | 1.000 | 0.312 | 0.312 | 0.900 | 0.357 | 0.257 | 0.963 | 0.457 | 0.420 |
| -0.521 | 0.933 | 0.312 | 0.245 | 0.875 | 0.357 | 0.232 | 0.944 | 0.457 | 0.402 |
| -0.508 | 0.933 | 0.321 | 0.254 | 0.875 | 0.369 | 0.244 | 0.944 | 0.471 | 0.416 |
| -0.493 | 0.933 | 0.330 | 0.264 | 0.875 | 0.381 | 0.256 | 0.944 | 0.486 | 0.430 |
| -0.460 | 0.867 | 0.330 | 0.197 | 0.850 | 0.381 | 0.231 | 0.926 | 0.486 | 0.412 |
| -0.414 | 0.867 | 0.339 | 0.206 | 0.825 | 0.381 | 0.206 | 0.907 | 0.486 | 0.393 |
| -0.386 | 0.867 | 0.349 | 0.215 | 0.825 | 0.393 | 0.218 | 0.907 | 0.500 | 0.407 |
| -0.359 | 0.867 | 0.358 | 0.224 | 0.825 | 0.405 | 0.230 | 0.907 | 0.514 | 0.422 |
| -0.321 | 0.867 | 0.367 | 0.234 | 0.825 | 0.417 | 0.242 | 0.889 | 0.514 | 0.403 |
| -0.296 | 0.867 | 0.376 | 0.243 | 0.825 | 0.429 | 0.254 | 0.870 | 0.514 | 0.385 |
| -0.285 | 0.867 | 0.385 | 0.252 | 0.825 | 0.440 | 0.265 | 0.870 | 0.529 | 0.399 |
| -0.238 | 0.867 | 0.394 | 0.261 | 0.825 | 0.452 | 0.277 | 0.870 | 0.543 | 0.413 |
| -0.193 | 0.867 | 0.404 | 0.270 | 0.825 | 0.464 | 0.289 | 0.870 | 0.557 | 0.428 |
| -0.185 | 0.867 | 0.413 | 0.280 | 0.800 | 0.464 | 0.264 | 0.852 | 0.557 | 0.409 |
| -0.172 | 0.867 | 0.422 | 0.289 | 0.800 | 0.476 | 0.276 | 0.852 | 0.571 | 0.423 |
| -0.159 | 0.867 | 0.431 | 0.298 | 0.800 | 0.488 | 0.288 | 0.852 | 0.586 | 0.438 |
| -0.154 | 0.867 | 0.440 | 0.307 | 0.800 | 0.500 | 0.300 | 0.852 | 0.600 | 0.452 |
| -0.150 | 0.867 | 0.450 | 0.316 | 0.800 | 0.512 | 0.312 | 0.852 | 0.614 | 0.466 |
| -0.139 | 0.867 | 0.459 | 0.325 | 0.800 | 0.524 | 0.324 | 0.852 | 0.629 | 0.480 |
| -0.091 | 0.867 | 0.468 | 0.335 | 0.800 | 0.536 | 0.336 | 0.852 | 0.643 | 0.495 |
| -0.053 | 0.867 | 0.477 | 0.344 | 0.800 | 0.548 | 0.348 | 0.852 | 0.657 | 0.509 |
| -0.049 | 0.867 | 0.486 | 0.353 | 0.775 | 0.548 | 0.323 | 0.833 | 0.657 | 0.490 |
| -0.042 | 0.800 | 0.486 | 0.286 | 0.750 | 0.548 | 0.298 | 0.815 | 0.657 | 0.472 |
| -0.032 | 0.800 | 0.495 | 0.295 | 0.750 | 0.560 | 0.310 | 0.815 | 0.671 | 0.486 |
| -0.016 | 0.800 | 0.505 | 0.305 | 0.750 | 0.571 | 0.321 | 0.815 | 0.686 | 0.501 |
| -0.007 | 0.733 | 0.505 | 0.238 | 0.725 | 0.571 | 0.296 | 0.796 | 0.686 | 0.482 |
| 0.008 | 0.733 | 0.514 | 0.247 | 0.700 | 0.571 | 0.271 | 0.778 | 0.686 | 0.463 |
| 0.025 | 0.733 | 0.523 | 0.256 | 0.700 | 0.583 | 0.283 | 0.778 | 0.700 | 0.478 |
| 0.040 | 0.733 | 0.532 | 0.265 | 0.700 | 0.595 | 0.295 | 0.759 | 0.700 | 0.459 |
| 0.055 | 0.733 | 0.541 | 0.275 | 0.700 | 0.607 | 0.307 | 0.759 | 0.714 | 0.474 |
| 0.078 | 0.667 | 0.541 | 0.208 | 0.675 | 0.607 | 0.282 | 0.741 | 0.714 | 0.455 |
| 0.120 | 0.667 | 0.550 | 0.217 | 0.675 | 0.619 | 0.294 | 0.722 | 0.714 | 0.437 |

PI: prognostic index

**Supplementary Table S3** Survival time and survival rate by PI-5 grade: grade 1 *vs.* grade 2

| Time | Variables | N | Survival time* | Survival Rate (%) | | | *P^a^* value |
| --- | --- | --- | --- | --- | --- | --- | --- |
|  |  |  |  | One year | Three year | Five year |  |
| One year | Grade1 | 54 | 93.30±2.31 | 96 | 96 | 96 |  |
|  | Grade2 | 70 | 73.58±3.87 | 83 | 81 | 81 | 0.012 |
| Three year | Grade1 | 54 | 85.39±3.70 | 85 | 85 | 85 |  |
|  | Grade2 | 70 | 55.77±4.41 | 83 | 54 | 54 | <0.001 |
| Five year | Grade1 | 54 | 85.77±3.59 | 85 | 85 | 85 |  |
|  | Grade2 | 70 | 45.52±3.92 | 83 | 49 | 35 | <0.001 |

PI: prognostic index

*Survival time was shown as mean±SD

**Supplementary Table S4** Predictive performance for 1-year, 3-year, and 5-year survival: PI-5 grade, TNM and CPI

| Time | Variables | AUC | SE | 95%CI | DIF | DIF Rate | *P^a^* | *P^b^* |
| --- | --- | --- | --- | --- | --- | --- | --- | --- |
| 1Year | TNM | 0.634 | 0.066 | 0.541~0.720 |  |  |  |  |
|  | PI-5 grade | 0.676 | 0.052 | 0.585~0.759 | 0.042 | 6.62% | 0.632 |  |
|  | CPI | 0.723 | 0.057 | 0.633~0.800 | 0.089 | 14.04% |  | 0.138 |
| 3Year | TNM | 0.611 | 0.049 | 0.517~0.698 |  |  |  |  |
|  | PI-5 grade | 0.681 | 0.043 | 0.590~0.763 | 0.071 | 11.62% | 0.242 |  |
|  | CPI | 0.719 | 0.047 | 0.629~0.797 | 0.108 | 17.68% |  | 0.013 |
| 5Year | TNM | 0.637 | 0.048 | 0.544~0.723 |  |  |  |  |
|  | PI-5 grade | 0.760 | 0.037 | 0.674~0.834 | 0.123 | 19.31% | 0.036 |  |
|  | CPI | 0.801 | 0.040 | 0.718~0.868 | 0.164 | 25.75% |  | <0.001 |

PI: prognostic index

CPI; combined predictor index

AUC: area under the ROC curve

DIF: difference of AUC between PI-5 grade, TNM and CPI

DIF Rate: percentage change of AUC between PI-5 grade, TNM and CPI

*^a^*: comparison of AUC by PI-5 grade and TNM in predicting 1-year, 3-year and 5-year survival

*^b^*: comparison of AUC by CPI and TNM in predicting 1-year, 3-year and 5-year survival

**Supplementary Table S5** Improvement effects of PI-5 grade for predicting 1-year, 3-year, and 5-year survival

|  | Point estimate^1^ | Point estimate^2^ | cfNRI^*^（95%CI） | *p* |
| --- | --- | --- | --- | --- |
| 1 Year | 80.0% | 50.5% | 0.295（0.040~0.484） | 0.010 |
| 3 Year | 81.6.% | 42.5% | 0.391（0.220~0.536） | <0.001 |
| 5 Year | 84.9% | 38.5% | 0.464（0.196~0.639） | 0.010 |
