# Supplementary figures and images for: Prognosis Prediction of Colorectal Cancer Using Gene Expression Profiles
Source: Front Oncol. 2019 Apr 9;9:252. doi: 10.3389/fonc.2019.00252 (PMC6465763; doi:10.3389/fonc.2019.00252)

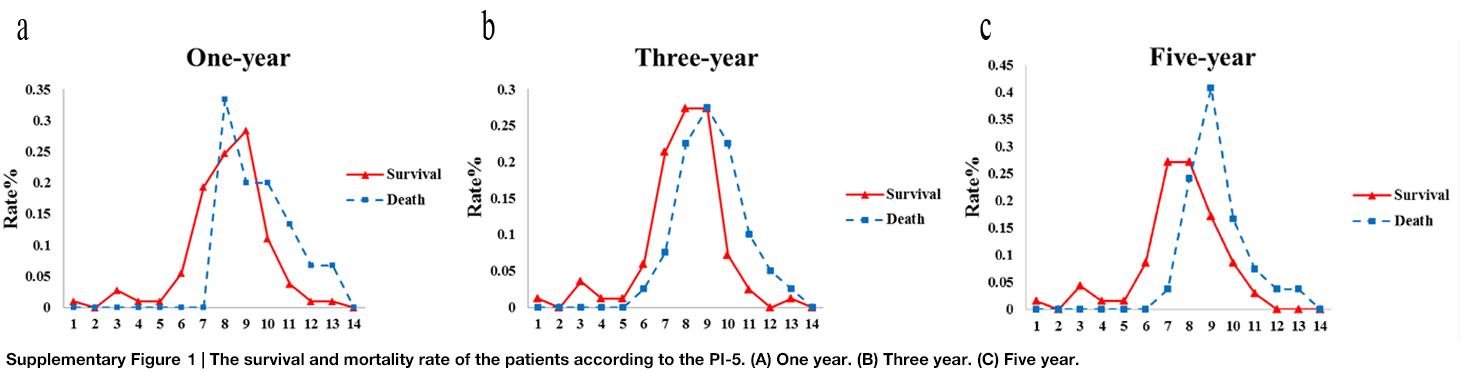

Supplement: Supplementary file 2 [file Image_1.JPEG]
